# Animal Ethical Views and Perception of Animal Pain in Veterinary Students

**DOI:** 10.3390/ani8120220

**Published:** 2018-11-23

**Authors:** Anna Valros, Laura Hänninen

**Affiliations:** Research Centre for Animal Welfare, Department of Production Animal Medicine, Faculty of Veterinary Medicine, University of Helsinki, 00014 Helsinki, Finland; anna.valros@helsinki.fi

**Keywords:** veterinary student, animal ethics, pain perception, animal, animal welfare

## Abstract

**Simple Summary:**

Veterinary students face several ethical challenges during their curriculum. We surveyed the animal ethical views of Finnish veterinary students, and also asked them to score the level of pain perception in different animal species. We found that the appreciation of pain perception of different animal species, and especially of those taxonomically further away from humans, appeared to increase during Finnish veterinary education. This implies that knowledge is important in improving views towards capacities of animals of varying taxa. Finnish veterinary students have a clear domination of utilitarian views in animal ethics, and veterinary education appeared to influence their views only to a small degree. We suggest that understanding the ethical views of veterinary students enables better planning of educational activities, to ensure that the students gain a good understanding of the potential variety of, and differences in, ethical views they will encounter as future professionals.

**Abstract:**

Veterinary students face several ethical challenges during their curriculum. We used the Animal Ethics Dilemma to study animal ethical views of Finnish veterinary students, and also asked them to score the level of pain perception in 13 different species. Based on the 218 respondents, the utilitarian view was the dominating ethical view. Mammals were given higher pain scores than other animals. The proportion of the respect for nature view correlated negatively, and that of the animal rights view positively, with most animal pain scores. Fifth year students had a higher percentage of contractarian views, as compared to 1st and 3rd year students, but this might have been confounded by their age. Several pain perception scores increased with increasing study years. We conclude that the utilitarian view was clearly dominating, and that ethical views differed only slightly between students at different stages of their studies. Higher pain perception scores in students at a later stage of their studies might reflect an increased knowledge of animal capacities.

## 1. Introduction

Veterinary students face several ethical challenges during their curriculum [1,2]. Distinguishing their own view on animal ethics and how it develops over time may help veterinarians to cope with difficult situations and emotions [3]. Ethically motivated decision-making is also important for client communication, for improving job satisfaction and for maintaining a positive public profile [4].

The professional development during veterinary education may lead to contrasting changes in empathy towards, and pain assessment of non-human animals. As part of the veterinary curriculum, the students are taught about non-human animal cognition and pain perception. They also gain insights in the complexity of animal-related issues in the society, such as those related to the food production chain and to animal use in research.

The effect of experience and age on the perception of animal pain is not straightforward. Younger veterinarians have been shown to rate pain higher, and to treat pain more [5]; to show no difference in pain ratings [6]; or to show lower pain ratings [7], as compared to older veterinarians. Further, empathy scores have been shown to decline during the course of education in both human doctors [8,9] and veterinarians [10] or not to change [6]. Despite these contradictory results, in general, knowledge is a prerequisite for a proper pain management [7,11,12,13] and thus, to improve veterinary education, we believe it is important to understand how veterinary students develop their views of animal pain perception during their studies.

In many countries there is no faculty-wide agreement on animal ethics, beyond legislation [14], which is also true for Finland. In addition, there is no internationally agreed view on which contents are best suited for veterinary ethics [14,15]. At the University of Helsinki, we aim at equipping students with both a systematic understanding of their own animal ethics views, and knowledge of the variation of ethical views, which they may encounter, rather than teaching a single ethical standard. Our approach is thus similar to that recommended by Clarkeburn [16]. At the University of Helsinki, which is the only University in Finland with a Veterinary Faculty, veterinary ethics is taught during the preclinical studies, which includes the first three years of the veterinary curriculum. Veterinary ethics is integrated into other subjects including several courses with elements of importance for the development of animal ethics. These include subjects such as pharmacology, where students learn about animal pain perception; animal welfare; and physiology, including e.g. stress physiology. During the clinical studies a one-day workshop on animal ethics and client communication is held as a part of clinical rounds in year 5. Teaching of veterinary and animal legislation is integrated into the studies throughout the entire 6-year period, but most teaching is provided during years 4 to 6.

Animal Ethics Dilemma ([17], www.aedilemma.net, AED) is an internet tool developed to test the proportion (%) of common animal ethical views one may have. AED has questions for utilitarian (roughly defined as ethical judgements being based on a strive to maximize the human and non-human animal welfare), animal rights (animals have inherent value that should be respected), respect for nature (a duty to protect the integrity of each species), contractarian (only indirect ethical obligations towards animals, because they can matter to other humans) and relational views (the moral status of an animal is defined by the relationship to the animal).

As a part of developing our teaching on animal ethics, we collected data on student views on animal ethics with AED and on perception of pain in different animal species. Our aim was to study which animal ethical views dominate among Finnish veterinary students. Further, we explored how students rate pain perception in non-human animals from different taxa, and if these views and ratings change during the veterinary education.

## 2. Materials and Methods

First, 3rd and 5th year students (2014–2016) at the Veterinary Faculty, University of Helsinki, Finland, were asked to evaluate their animal ethical views using the AED and to report these via an electronic questionnaire. In the questionnaire, students were further asked to score the level of pain perception using a Likert-scale from 0 (pain causes only reflexes) to 10 (pain is subjectively experienced) in 13 different animals, representing wild and domestic animals and vertebrate and invertebrate animals of a wide range (see S1 for the questionnaire).

Background factors collected via the electronic questionnaire included gender, study year and year of birth. The replies were anonymous, and participation was voluntary.

The investigations were carried out following the rules of the Declaration of Helsinki of 1975. The work is in compliance with the guidelines of the Finnish national board of research integrity (TENK; http://www.tenk.fi/en/tenk-guidelines) according to which no ethical review was required.

### 2.1. Student Demographics

We obtained a total of 218 answers, which represents a response rate of approximately 35 %. The distributions of respondents of different study years over the three response years are presented in Table 1. The data included students from at least two response years for each study year, and each student replied to the questionnaire only once. The age of the students ranged from 19 to 44 years (on average 25 years). In total 21 of the respondents were male (9.6 %) which is equal to the proportion of male students in the faculty in 2016. Due to the small number of male students the effect of gender was not analyzed.

### 2.2. Data Handling and Statistical Analyses

For statistical analyses, the students were classified into two more or less equal age groups: ‘older’ (24 years or older, *n* = 104) and ‘younger’ (under 24 years, *n* = 112). Two respondents had not indicated their age. Age and study year were clearly associated with each other: 98% (60/61) of the 5th year students (average age 27.6 years) fell into the ‘older’ age class. The corresponding numbers were 40% (15/38) and 32% (37/117) for 3rd (average age 24.3 years) and 1st year students (average age 23.6 years), respectively.

Due to non-normal distribution of the data we tested differences between study years with Kruskall-Wallis tests, followed by Bonferroni-corrected pairwise tests when relevant, and between age classes (‘older’ and ‘younger’) with Mann-Whitney U tests. Correlations within and between the proportions of different ethical views from AED and animal pain scores were tested with Spearman rank correlations.

## 3. Results

### 3.1. Overall Results: Animal Ethical Views

In general, based on the replies to the AED, the utilitarian view was the dominating ethical view. Both the utilitarian and the respect for nature views were represented on some level in almost all the answers, while the utilitarian view got a much higher median percentage. Animal rights, relational and contractarian views were represented in a lower proportion of the answers but did occur to a significant degree in some of these (see Table 2).

The proportion of utilitarian view in the AED results correlated negatively with the proportion of all other ethical views (r_s_ = −0.26 to −0.54, *p* < 0.001 for all). In addition, the proportion of animal rights view correlated negatively with the proportion of the respect for nature view (r_s_ = −0.26, *p* < 0.001). No further correlations between the proportions of different animal ethical views were found (*p* > 0.05 for all).

### 3.2. Overall Results: Animal Pain Perception Scores

Overall descriptive results for the pain perception scores given by the students are presented in Table 3. Chimpanzees were scored the highest, while earthworms were given the lowest score. Pain perception scores could be numerically classified into three groups: the highest scoring group included all mammals as well as the budgerigar (median score 10), the intermediately scoring group included the fish and the invertebrate octopus (7) and the lowest scoring group included all other invertebrates (3–4).

Pain scores of almost all different animals correlated positively with each other (r_s_ = 0.18 to 0.93, *p* < 0.01 for all), however, the chimpanzee pain score did not correlate with the pain score given to the fly or the earthworm (*p* > 0.1 for both).

### 3.3. Correlations between Animal Ethical Views and Pain Perception Scores

We found several statistically significant, though rather low correlation coefficients: The proportion of the respect for nature view in the AED correlated negatively (r_s_ = −0.16 to −0.25, *p* < 0.05 for all) and that of the animal rights view positively (r_s_ = 0.15 to 0.36, *p* < 0.05 for all) with all animal pain scores, except for the chimpanzee score (*p* > 0.1 for both). The proportion of these two ethical views also correlated with the average pain perception score (respect for nature; r_s_ = −0.3 and animal rights; r_s_ = 0.3, *p* < 0.001 for both). There was no correlation between the proportion of utilitarian, contractarian or relational view and any of the pain perception scores (*p* > 0.1 for all).

The proportion of contractarian view, according to the AED, differed between students of different study years (Kruskall–Wallis χ^2^(2) = 11.9, *n* = 213, *p* < 0.01). Fifth year students had a higher percentage of contractarian view in AED (Median: 0; Min–max: 0–67), as compared to 1st (0; 0–17, *p* < 0.01) and 3rd year (0; 0–17, *p* < 0.05) students. Correspondingly, ’older’ students tended to have a higher percentage of contractarian view in AED than ‘younger’ students (0; 0–67 and 0; 0–33, respectively, Mann-Whitney U: 6000, *n* = 211, *p* < 0.1).

Several pain perception scores, as well as the average pain perception score, increased with increasing study year (Table 3). Of the pain perception scores, only the budgerigar score was affected by age, with ‘younger’ respondents scoring lower scores than ‘older’ ones (9; 2–10 vs. 10; 3–10, Mann-Whitney U = 6363, *n* = 211, *p* < 0.05). In addition, the dolphin score tended to be lower for ‘younger’ than ‘older’ students (10; 5–10 vs. 10; 6–10, Mann-Whitney U = 6215, *n* = 212, *p* = 0.05)

## 4. Discussion

The animal ethical view estimated using the Animal Ethics Dilemma of Finnish veterinary students, was dominated by a utilitarian view throughout the study years. The veterinary students scored animal pain perception higher the further they had advanced in their studies, which appeared to be rather independent of their age.

The strong utilitarian view domination in this study might be due to the utilitarian view being more easily combined with a veterinary education, where some level of animal instrumentalization is unavoidable. Lund and others (2016) [18] studied ethical views of a general selection of meat eaters, vegetarians and vegans in the UK, and showed a more even distribution between the animal rights view and the utilitarian view than we report in this study. In addition, they showed that the animal rights view dominated in the vegetarians and vegans, while meat eaters were more utilitarian. As far as we know, however, there are no similar studies on animal ethical views on broader samples of the Finnish population, so we cannot exclude that the currently presented results are not merely a reflection of the attitudes of the general Finnish population. In a Finnish nationwide study Kupsala et al (2015) [19] reported an average rating of animal instrumentalization as 2.25 (SD 1.18) on a scale from 1–5, where 5 meant that the respondents totally agreed with the statement ‘An animal should be seen primarily as a means of production’. Thus, a certain degree of utilitarian animal ethics was apparent also in the general public, but with quite a large variation. It is also worth mentioning that even though some EU-countries have implemented a non-kill-policy regarding healthy companion animals [20], this is not the case in Finland. As allowing euthanasia of healthy animals is typically against the view of animal rights advocates [21], this suggests that also in general the animal rights view might not be as strong in Finnish society as in at least some other European countries.

The animal ethics dilemma was originally developed for teaching [17] while components of it has been used also in research [18], even though it has not been formally validated for research purposes. It is probable that the tool simplifies the evaluation of animal ethical views, partly due to it´ s forced-choice design [18] and as the respondents can chose only one answer, representing one ethical view, per question. The results regarding animal ethical views should thus be considered with some care.

Pain perception was scored the highest in mammals, followed by other vertebrates, and the lowest in invertebrates. This corresponds to pain ratings in the nationwide survey performed in Finland by Kupsala and others (2015) [19] where mammals were assessed to experience pain much more often than salmon and shrimp. Also, Phillips and McCulloch (2005) [22] found that students of different nationalities ascribed mammals as having higher levels of sentience than chicken and fish. The respondents in our study showed a higher variation in their rating of pain perception for non-mammals than mammals. A similarly higher uncertainty of pain rating for non-mammalian (chicken, salmon and shrimp), as compared to mammalian animals, was seen in the study by Kupsala and others (2015) [19].

The fact that the animal rights view correlated positively with pain perception scorings is not surprising. Previous studies have similarly shown that animal sentience is given a higher attribute by students applying more constrains on animal use [22], and that a less instrumental view on animals is correlated to a higher belief in animal mind [19]. We find it more difficult to speculate why the respect for nature view correlated negatively with pain perception scores, but this might be related to accepting pain as a part of ‘natural life’. Lund and others (2016) [18] reported that meat eaters had a higher level of the respect for nature view as compared to vegans and vegetarians, which might indicate a generally less critical view of animal use. This correlation might, however, merely be due to the fact that the proportion of animal rights and respect for nature views correlated negatively in our study.

Pain perception scoring for chimpanzees appeared to be high irrespective of ethical view and slightly independent of pain perception in other species. Interestingly, Phillips and McCulloch (2005) [22] found that even though nationality had an effect on several attitudes of students towards animals, students of different nationalities did not differ in how they evaluated the level of sentience of monkey, dog and fox. It might therefore be that the perception of animals perceived to be culturally closer to humans, are less affected by other attitudes. Phillips and McCulloch (2005) [22] included ‘new-born baby’ in their list of animals to be assessed for level of sentience, and the students in their study scored the sentience level of a baby lower than that of a monkey or a dog. We only included non-human animals in our questionnaire, which does not allow us to estimate how our students would have rated human pain perception. Also, it is possible that the results would have changed if the students had related their ratings to humans.

It should be noted that the pain perception scale we used was a commonly used simple 11-point Numerical Rating Scale [6,7,23] and could have been somewhat confusing, as it assumed the students somehow understood the link between concepts of pain perception and the level of consciousness or mental abilities of the animals. This link, however, is a rather accepted issue in science. It has been suggested that while nociception is a mere sensory ability, pain perception is primarily a subjective feeling (for a review, see eg Sneddon and others [19]).

Students in later years of their studies (esp. 5th year) appeared to have more knowledge about animal pain perception, especially regarding species that were scored lower in general, such as most invertebrates. These are species often regarded as having a lower moral standing in society [19], which is reflected also in some pieces of legislation. One example is the EU directive (2010/63/EU) on the protection of animals used for scientific purposes, which does not include most invertebrates: *‘In addition to vertebrate animals including cyclostomes, cephalopods should also be included … as there is scientific evidence of their ability to experience pain, suffering, distress and lasting harm’*. The students therefore might enter the veterinary curriculum with a more ‘popular’ view of animal pain, and then develop this by gaining more information during their education. Also, the scoring of fish and birds was higher in students a few years into their curriculum, which might reflect that these taxa are still somewhat underestimated regarding their cognitive abilities. This could be explained by the fact that the empathic response is amplified by similarity and familiarity [24,25,26]. Fish in particular are often treated in ways that would never be accepted for mammals; some species are, for example, slaughtered slowly by suffocating to death or bled without stunning [27], while an increasing body of evidence shows that fish actually perceive pain at a level comparable to mammals [28].

Further, as pain perception scores were affected by age of the students to a very small degree (only the score for budgerigar), the difference in the scoring between students at different stage in their curriculum, might reflect an improvement in the level of knowledge of pain perception, acquired during their veterinary education. Surprisingly, also the pain perception score of cattle was higher in 5th year students than in 1st year students, and production animals (cattle and pigs) scored more or less as high as cats. Related findings from North American Veterinary students showed, that the students were likely to assess farm animals to be less sentient or having lower cognitive processes than dogs and cats [29]. Also, a current study showed that Italian veterinary students estimated the freedom to express normal species-specific behaviors and the freedom from fear and distress less important for the welfare of livestock animals than to pets [30]. Further, Levine and others (2005) [29] and Kupsala and others (2015) [19] showed that dogs were more frequently assessed to be able to feel pain than farm animals, such as cows and pigs.

We could not see indications of the commonly reported effect of reduced perception of pain [31] and empathy in the course of medical education [9,10]. Instead, our results are in line with previous findings among Finnish veterinary students and graduated veterinarians on animal empathy and attitudes towards treating disbudding pain in calves [6]. In this study however, we did not test empathy as such, and it is possible, that the rating of pain perception used in this study merely tested the level of biological knowledge of other species. This study would be interesting to repeat in different countries, as nationality appears to affect attitudes to animals [22]. It would also be interesting to be able to compare multiple veterinary faculties within one country to evaluate a possible effect of faculty attitudes on the students´ perceptions.

Students in their 5th study year were somewhat more contractarian in their ethical views than students in earlier years. This, however, might be confounded by the fact that students of older age have more experience of life in general. We suggest this could be due to students usually entering the veterinary faculty with a rather low knowledge level regarding food producing animals, and animal use in general (*personal observation by the authors*), and as they learn more about the regulations and commonly accepted practices affecting and constraining animal husbandry, this might be reflected in increased contractarian views. In line with our findings, senior Swedish veterinary students were more likely to accept the use of animals in experiments than younger ones [32]. The Swedish veterinary students explained their attitude change to be due to greater knowledge, a better understanding of the necessity, more accepting and tolerant views, and a less prejudiced view of the use of animals.

## 5. Conclusions

The animal ethics views of Finnish Veterinary students are typically dominated by a utilitarian view, and that students at a later stage of their curriculum have a larger proportion of contractarian view than students in earlier years. The increased appreciation of animal pain perceptions in students further on in their education probably reflects increased knowledge of animal capacities, gained through the ongoing veterinary education.

## Figures and Tables

**Table 1:** Distribution of students replying to the survey (218 in total), according to year of study and response year.

Response Year	Year of Study		
	1	3	5
2014	66	19	0
2015	25	19	45
2016	28	0	16
Total	119	38	61

**Table 2:** Overall animal ethical views of Finnish veterinary students based on the Animal Ethics Dilemma (*n* = 218).

Animal Ethical Views	Median	Min	Max	Answers with 0%, % ^1^
Utilitarian	50	0	92	4
Respect for nature	25	0	67	3
Animal rights	17	0	92	15
Relational view	2	0	42	49
Contractarianism	0	0	67	86

^1^ percentage of respondents for which the respective animal ethical view was not represented at all in the AED (Animal Ethics Dilemma) results.

**Table 3:** Animal pain perception scores given by veterinary students (*n* = 213), overall, and in study years 1, 3 and 5, based on a scale from 0: pain causes only reflexes–10: pain is subjectively experienced. Results are given as median (min-max). P-values indicate a difference between study years (n.s. for *p* > 0.1 and #, *, **, for *p* < 0.1, *p* < 0.05 and *p* < 0.01, respectively).

Animal	Overall Median (Min–Max)	1st Year *n* = 119	3rd Year *n* = 38	5th Year *n* = 61	Sign. Level
Chimpanzee	10 (7–10)	10 (8–10)	10 (7–10)	10 (8–10)	n.s.
Dolphin	10 (5–10)	10 (5–10)	10 (5–10)	10 (8–10)	n.s.
Cat	10 (5–10)	10 (5–10)	10 (7–10)	10 (8–10)	#
Pig	10 (6–10)	10 (6–10)	10 (6–10)	10 (8–10)	n.s.
Lion	10 (5–10)	10 (5–10)	10 (7–10)	10 (8–10)	n.s.
Cattle	10 (5–10)	10(5–10) ^a^	10 (5–10) ^ab^	10(7–10) ^b^	*
Budgerigar	10 (2–10)	9 (2–10) ^a^	10 (3–10) ^b^	10(5–10) ^b^	**
Octopus	7 (1–10)	7 (1–10)	8 (1–10)	7 (2–10)	n.s.
Fish	7 (1–10)	6 (1–10) ^a^	8 (1–10) ^b^	7 (2–10) ^b^	**
Bee	4 (1–10)	3 (1–10) ^a^	5 (1–10) ^ab^	6 (1–10) ^b^	*
Spider	4 (0–10)	3 (0–10) ^a^	4 (1–10) ^ab^	5 (0–10) ^b^	*
Fly	3 (0–10)	3 (0–10) ^a^	4 (1–10) ^ab^	4 (1–10) ^b^	*
Earthworm	3 (0–10)	2 (0–10) ^a^	4.5 (1–10) ^b^	4 (1–10) ^b^	*
Average *	7.5 (3.3–10)	7.2 (3.3–10) ^a^	7.7 (4.8–10) ^ab^	7.8 (5.5–10) ^b^	**

* average (min-max) pain perception score for all animals; ^a, b^ Values within rows lacking common superscript letters differ.

## Supplementary Materials

The following are available online at http://www.mdpi.com/2076-2615/8/12/220/s1, S1: The questionnaire. (Due to the data containing data, such as year of birth and gender, based on which individuals might be identifiable, the data is only supplied on request).

## References

[1] Herzog H.A., Vore T.L., New J.C. (1989). Conversations with Veterinary Students: Attitudes, Ethics, and Animals. Anthrozoos.

[2] Verrinder J.M., Phillips C.J.C. (2014). Identifying Veterinary Students’ Capacity for Moral Behavior Concerning Animal Ethics Issues. J. Vet. Med. Educ..

[3] Magalhães-Sant’Ana M. (2016). A theoretical framework for human and veterinary medical ethics education. Adv. Health Sci. Educ..

[4] Mullan S., Main D. (2001). Principles of ethical decision-making in veterinary practice. In Pract..

[5] Raekallio M., Heinonen K.M., Kuussaari J., Vainio O. (2003). Pain Alleviation in Animals: Attitudes and Practices of Finnish Veterinarians. Vet. J..

[6] Norring M., Wikman I., Hokkanen A., Kujala M.V., Hänninen L. (2014). Empathic veterinarians score cattle pain higher. Vet. J..

[7] Huxley J.N., Whay H.R. (2006). Current attitudes of cattle practitioners to pain and the use of analgesics in cattle. Vet. Rec..

[8] Hojat M., Mangione S., Nasca T.J., Rattner S., Erdmann J.B., Gonnella J.S., Magee M. (2004). An empirical study of decline in empathy in medical school. Med. educ..

[9] Neumann M., Edelhäuser F., Tauschel D., Fischer M.R., Wirtz M., Woopen C., Haramati A., Scheffer C. (2011). Empathy Decline and Its Reasons: A Systematic Review of Studies With Medical Students and Residents. Acad. Med..

[10] Hazel S.J., Signal T.D., Taylor N. (2011). Can Teaching Veterinary and Animal-Science Students about Animal Welfare Affect Their Attitude toward Animals and Human-Related Empathy?. J. Vet. Med. Educ..

[11] Paul E.S., Podberscek A.L. (2000). Veterinary education and students’ attitudes towards animal welfare. Vet. Rec..

[12] Hewson C.J., Dohoo I.R., Lemke K.A., Barkema H.W. (2007). Factors affecting Canadian veterinarians’ use of analgesics when dehorning beef and dairy calves. Can. Vet. J..

[13] Mich P.M., Hellyer P.W., Kogan L., Schoenfeld-Tacher R. (2010). Effects of a Pilot Training Program on Veterinary Students’ Pain Knowledge, Attitude, and Assessment Skills. J. Vet. Med. Educ.

[14] Magalhães-Sant’Ana M., Lassen J., Millar K.M., Sandøe P., Olsson I.A. (2014). Examining Why Ethics Is Taught to Veterinary Students: A Qualitative Study of Veterinary Educators’ Perspectives. J. Vet. Med. Educ..

[15] Magalhães-Sant’Ana M. (2014). Ethics teaching in European veterinary schools: S qualitative case study. Vet. Rec..

[16] Clarkeburn H. (2002). The aims and practice of ethics education in an undergraduate curriculum: Reasons for choosing a skills approach. J. Furth. High. Educ..

[17] Hanlon A.J., Algers A., Dich T., Hansen T., Loor H., Sandøe P. (2007). Animal Ethics Dilemma: An interactive learning tool for university and professional training. Anim. Welf..

[18] Lund T.B., McKeegan D.E.F., Cribbin C., Sandøe P. (2016). Animal Ethics Profiling of Vegetarians, Vegans and Meat-Eaters. Anthrozoös.

[19] Kupsala S., Vinnari M., Jokinen P., Räisänen P. (2015). Citizen Attitudes to Farm Animals in Finland: A Population-Based Study. J. Agric. Environ. Ethics.

[20] EU Animal Welfare Platform. Proceedings of the 2nd Meeting.

[21] Sandøe P., Christiansen S.B. (2008). Ethics of Animal Use.

[22] Phillips C.J.C., McCulloch S. (2005). Student attitudes on animal sentience and use of animals in society. J. Biol. Educ..

[23] Wikman I., Hokkanen A.-H., Pastell M., Kauppinen T., Valros A., Hänninen L. (2013). Dairy producer attitudes to pain in cattle in relation to disbudding calves. J. Dairy. Sci..

[24] de Waal F.B. (2008). Putting the altruism back into altruism: The evolution of empathy. Annu. Rev. Psycholo..

[25] Drwecki B.B., FAU M.C., FAU W.S., Prkachin K.M. (2011). Reducing racial disparities in pain treatment: The role of empathy and perspective-taking. Pain.

[26] Rae Westbury H., Neumann D.L. (2008). Empathy-related responses to moving film stimuli depicting human and non-human animal targets in negative circumstances. Biol. Psychol..

[27] Kestin S.C., Robb D.H.F., van de Vis J.W. (2002). Protocol for assessing brain function in fish and the effectiveness of methods used to stun and kill them. Vet. Rec..

[28] Sneddon L.U., Elwood R.W., Adamo S.A., Leach M.C. (2014). Defining and assessing animal pain. Anim. Behav..

[29] Levine F.D., Mills D.S., Houpt K.A. (2005). Attitudes of veterinary students at one US college toward factors relating to farm animal welfare. J. Vet. Med. Educ..

[30] Mariti C., Pirrone F., Albertini M., Gazzano A., Diverio S. (2018). Familiarity and Interest in Working with Livestock Decreases the Odds of Having Positive Attitudes towards Non-Human Animals and Their Welfare among Veterinary Students in Italy. Animals.

[31] Cheng Y., Lin C., Liu H., Hsu Y., Lim K., Hung D., Decety J. (2007). Expertise Modulates the Perception of Pain in Others. Curr. Biol..

[32] Hagelin J., Hau J., Carlsson H.E. (2000). Attitude of Swedish veterinary and medical students to animal experimentation. Vet. Rec..

