# Supplementary material for: Animal Ethical Views and Perception of Animal Pain in Veterinary Students
Source: Animals (Basel). 2018 Nov 23;8(12):220. doi: 10.3390/ani8120220 (PMC6315997; doi:10.3390/ani8120220)
Supplement: Supplementary file 1 [file animals-08-00220-s001.pdf]

animals

# Supplementary Materials: Animal Ethical Views and Perception of Animal Pain in Veterinary Students

**Anna Valros †, and Laura Hänninen*, †**

Research centre for animal welfare & Department of production animal medicine, Faculty of veterinary medicine, University of Helsinki, 00014 Helsinki, Finland; anna.valros@helsinki.fi

* Correspondence: laura.hanninen@helsinki.fi; Tel.: +358-50-415-1180

† These authors contributed equally to this work.

Received: 12 November 2018; Accepted: 20 November 2018; Published: 23 November 2018

## Animal welfare and ethics

Answering this questionnaire will take you only a few minutes. The aim is to increase our understanding of the ethical thinking, and perceptions of animal welfare, of veterinary students. Before filling in the questionnaire, please visit the Animal Ethics Dilemma — webpage (http://www.aedilemma.net/), do the test to evaluate your animal ethics views, and write down the results.

## Ethics

Give the relative distribution of your animal ethical views

| | Percentage: |
|---|---|
| Utilitarian | __ |
| Contractarian | __ |
| Relational | __ |
| Animal rights | __ |
| Respect for nature | __ |

## Pain perception

Animal sentience is under debate. Assess the animal´s pain perception on a scale from 1-10 (1: pain merely causes a reflex, 10: pain is a subjective experience)

| | Scale 1-10 |
|---|---|
| Lion | __ |
| Octopus | __ |
| Cat | __ |
| Bee | __ |
| Cattle | __ |
| Fish | __ |
| Budgerigar | __ |
| Earthworm | __ |
| Chimpanzee | __ |
| Dolphin | __ |
| Fly | __ |
| Pig | __ |
| Spider | __ |

## Backround information

| | |
|---|---|
| Year of birth (for example 1968) | __ |

animals MDPI

**Gender**

Female __

Male __

**Which year of study are you in**

1 __

2 __

3 __

4 __

5 __

6 __

over 6 __

*This is a translation of the original questionnaire distributed in Finnish*
